# Optimal Candidates to Do Fresh Embryo Transfer in Those Using Oral Contraceptive Pretreatment in IVF Cycles

**DOI:** 10.3389/fphys.2021.576917

**Published:** 2021-03-11

**Authors:** Yao Lu, Yichao Niu, Yuan Wang, Yaqiong He, Ying Ding, Xinyuan Lu, Bing Xu, Steven R. Lindheim, Yun Sun

**Affiliations:** ^1^Center for Reproductive Medicine, Ren Ji Hospital, School of Medicine, Shanghai Jiao Tong University, Shanghai, China; ^2^Shanghai Key Laboratory for Assisted Reproduction and Reproductive Genetics, Shanghai, China; ^3^Key Laboratory of Health Technology Assessment of Ministry of Heath, Collaborative Innovation Center of Social Risks Governance in Health, School of Public Health, Fudan University, Shanghai, China; ^4^Department of Obstetrics and Gynecology, Boonshoft School of Medicine, Wright State University, Dayton, OH, United States

**Keywords:** oral contraceptive, *in vitro* fertilization, fresh embryo transfer, GnRH antagonist, GnRH agonist

## Abstract

**Objective:**

Concern regarding the adverse impact of pretreatment of oral contraceptives (OC) prior to ovarian stimulation for *in vitro* fertilization (IVF) on pregnancy outcome has been debated. We investigated factors that may be associated with live birth rate (LBR) in fresh embryo transfer cycles after OC pretreatment.

**Methods:**

A retrospective study was conducted at the Reproductive Center of Ren Ji Hospital, Shanghai, China. 814 women aged 20–35 years undergoing their first autologous IVF cycle and fresh embryo transfer after OC pretreatment were included. Long gonadotropin releasing hormone (GnRH) agonist (a) or GnRH antagonist (ant) protocol was used for ovarian stimulation. Predictive factors for LBR were identified using multivariate logistic regression analysis.

**Results:**

Multivariate logistic regression analysis demonstrated that using GnRH-ant protocol for ovarian stimulation was associated with significantly lower LBR (OR 0.70, 95% CI 0.52–0.93), while endometrial thickness on day of hCG trigger was associated with increased LBR (OR 1.16, 95% CI 1.06–1.27). Despite comparable patients’ age, duration of infertility, BMI and basal FSH between GnRH-a and GnRH-ant groups, those using GnRH-ant resulted in significantly lower LBR compared to the GnRH-a group (37.4 vs. 48.5%, *p* = 0.002). Using ROC analysis and a cut-off endometrial thickness of < and ≥ 9.5 mm, those < 9.5 mm using GnRH-ant resulted in significantly lower LBR (28.5 vs. 43.4%, *p* = 0.004), while no differences were noted with an endometrial thickness ≥9.5 mm (49.6 vs. 51.1%, *p* = 0.78).

**Conclusions:**

Live birth was significantly impacted in OC pre-treated GnRH-ant cycles with an endometrial thickness of <9.5 mm on day of hCG trigger. Cryopreservation of all embryos in these cycles should be considered.

## Introduction

Oral contraceptives (OC) have been widely used to prevent pregnancy, and for the treatment of dysmenorrhea or menstrual disorders since being first approved in 1960 (Golobof and Kiley, 2016). With the increasing utilization of *in vitro* fertilization-embryo transfer (IVF-ET) world-wide, OC have extensively been employed as a pretreatment before controlled ovarian stimulation in IVF cycles (Farquhar et al., 2017).

OC can control the induction of spontaneous menses via the suppression of the basal follicle stimulating hormone (FSH) and luteinizing hormone (LH) levels, and thereby has become a mainstay approach for cycle scheduling (Ozgur et al., 2016). Previous data has suggested that lowering circulating gonadotropins with OC pretreatment may aide in the synchronization of follicular development and as a result optimize oocyte yield during ovarian stimulation (Keltz et al., 2007; Kim et al., 2009; Bozdag et al., 2012; Yildiz, 2015; Farquhar et al., 2017). However, recently there have been an increasing number of studies reporting the use of OC pretreatment may be potentially related to impaired pregnancy outcomes after fresh embryo transfers (Garcia-Velasco and Fatemi, 2015; Farquhar et al., 2017; Wei et al., 2017; Xu et al., 2019b; Lu et al., 2020). A recent Cochrane meta-analysis by Farquhar et al. which included six randomized controlled trials (RCTs) showed that OC pretreatment was associated with a lower ongoing pregnancy rate or live birth rate (LBR) after fresh embryo transfer than no pretreatment in gonadotropin releasing hormone (GnRH) antagonist (ant) cycles. In contrast, other retrospective studies reported that patients using OC pretreatment had similar probabilities of achieving a live birth compared to patients not-using OC following fresh embryo transfer in both GnRH-ant and GnRH agonist (a) protocols (Xu et al., 2019a; Montoya-Botero et al., 2020).

Concerns regarding the use of OC pretreatment in IVF cycles remain controversial as some would argue that even with these differences; the significance with respect to clinical outcomes is marginal. As such, cycle scheduling and synchronization remains necessary in IVF management. Thus, our study was conducted to investigate for predictive factors that may be associated with LBR in fresh embryo transfer cycles after OC pretreatment, and to identify optimal candidates who could be ideally considered for a fresh transfer after OC pretreatment for IVF cycles.

## Materials and Methods

This retrospective study was conducted at the Reproductive Center of Ren Ji Hospital affiliated to Shanghai Jiao Tong University School of Medicine (Shanghai, China). The study protocol was approved by the Ethics Committee for Reproductive Medicine of Ren Ji Hospital and conducted in compliance with the Declaration of Helsinki.

### Study Population

From January 2014 to June 2017, all patients aged 20–35 years with a basal FSH < 10 IU/L, and a menstrual cycle length of 21–35 days who were undergoing their first autologous IVF or intra-cytoplasmic sperm injection (ICSI) cycle followed by fresh embryo transfer were reviewed. All patients who used long GnRH-a or GnRH-ant protocol for ovarian stimulation after OC pretreatment were included. Excluded were cycles of those diagnosed with polycystic ovary syndrome (PCOS) according to Rotterdam Eshre/Asrm-Sponsored Pcos Consensus Workshop Group. (2004), uterine abnormalities (such as submucosal leiomyoma and congenital malformations), untreated hydrosalpinges, and those undergoing pre-implantation genetic testing (PGT).

### Stimulation Protocol

Utilization of OC pretreatment was started on cycle day 2–5 following menstruation and taken daily for 17–21 consecutive days. Three types of low-dose monophasic combined OC were prescribed by physicians including cyproterone acetate/ethinylestradiol (Diane-35, Bayer Vital GmbH), desogestrel/ethinylestradiol (Marvelon, MSD) and drospirenone/ethinylestradiol (Yasmin, Bayer Vital GmbH).

Ovarian stimulation using daily injections of 150–300 IU/day of recombinant FSH (Gonal-F, Merck Serono, Geneva, Switzerland) and/or urinary human menopausal gonadotropin (hMG) (Menopur, Ferring, Switzerland) was individualized based on age, body mass index (BMI), and ovarian reserve. For those using GnRH-a, Triptorelin (0.05 mg daily, Ferring) was administered on the 14–15th day of OC administration for 10–14 days. In those using GnRH-ant, Ganirelix (0.25 mg daily, Vetter Pharma-Fertigung GmbH&Co. KG or Cetrorelix, Merck Serono) was initiated when lead follicle growth exceeded 12 mm in size. All cycles were monitored starting on stimulation days four to five, and dosing was adjusted accordingly by serum hormone levels and transvaginal sonography. When lead follicles reached ≥18 mm diameter in size, recombinant hCG 250 mcg (Ovitrelle, Merck Serono) was administered to induce final follicle maturation. Thirty-six hours later, transvaginal oocyte retrieval was scheduled.

### IVF and Embryo Transfer

Retrieved eggs were fertilized by IVF unless ICSI was indicated. Indications for ICSI include oligozoospermia (sperm concentration: <15 × 10^6^/ml)/asthenozoospermia [progressive motility rate < 32% or total motility rate (progressive and non-progressive motility rate) < 40%]/teratozoospermia (normal morphology rate < 4%), unexplained infertility and non-obstructive azoospermia. Semen analysis and evaluation of sperm morphology was based on *WHO Laboratory Manual for the Examination and Processing of Human Semen* (5th Edition) (Lu and Gu, 2010). Fertilization was checked 16–18 h later. Embryos were cultured to cleavage stage (this was the standard during the time period of our study). Cleavage embryos with at least 6 cells and less than 20% fragmentation on day 3 were defined as good quality and eligible for transfer. Up to two embryos were transferred transcervically three days post-retrieval.

Embryo transfers were performed using soft plastic catheters (Wallace, Smith Medical International Ltd., United Kingdom) using the after load technique under ultrasound guidance. The luteal phase was supported using the vaginal progesterone gel (Crinone 90 mg daily, Merck Serono) and oral dydrogesterone (10 mg, twice daily, Abbott), starting the day following oocyte retrieval until 10 weeks of gestation if pregnancy was confirmed. Surplus good quality cleavage embryos were cryopreserved on day three. Embryos of poor quality on day three were cultured for blastocysts and cryopreserved if they scored ≥ 4BC according to the Gardner criteria (Matsuura et al., 2010).

### Measured Outcomes

The primary aim of this study was to identify predictive factors for LBR following fresh embryo transfer, which was defined as a delivery of a life neonate at ≥28 gestational weeks. Pregnancy outcomes included clinical pregnancy rate (CPR), early pregnancy loss rate (EPLR) and twin LBR were also measured. Clinical pregnancy was defined as presence of gestational sac at 6–8 weeks of gestation. Early pregnancy loss was defined as spontaneous pregnancy loss before 12 weeks gestation.

### Statistical Analysis

Data collected included patient’s age, BMI, basal hormone levels FSH, LH, and estradiol (E2). Cycle stimulation characteristics included GnRH-a or GnRH-ant use, total gonadotropin (Gn) dose, days of stimulation, endometrial thickness, number of oocytes retrieved, number of fertilized oocytes, embryos transferred/cryopreserved, and pregnancy outcomes.

Continuous variables were expressed as mean (M) and standard deviation (*SD*), while categorical variables were presented as frequency (*n*) and percentage (%). The distribution of normality was tested by Shapiro-Wilk test, and differences between groups were compared by student’s *t*-test, while the distribution of categorical variables was compared by chi-square test. Stepwise logistic regression was applied to identify significant predictive factors for live birth rate after fresh embryo transfer. The significance level of the candidate factors was set to 0.05 to enter the model and 0.10 to stay in the model. Results of the logistic regression analysis were expressed as odds ratio (OR) and 95% confidence interval (CI). Receiver operating characteristic (ROC) curves were calculated and the area under the curves (AUC) was used to assess the discriminative power of the regression model, and to identify the optimal cut-off value using the Youden index. Two-sided alpha level of 0.05 was considered as statistically significant. SPSS 21.0 (SPSS Inc., Chicago, IL, United States) was used for all statistical analyses.

## Results

**FIGURE 1:**
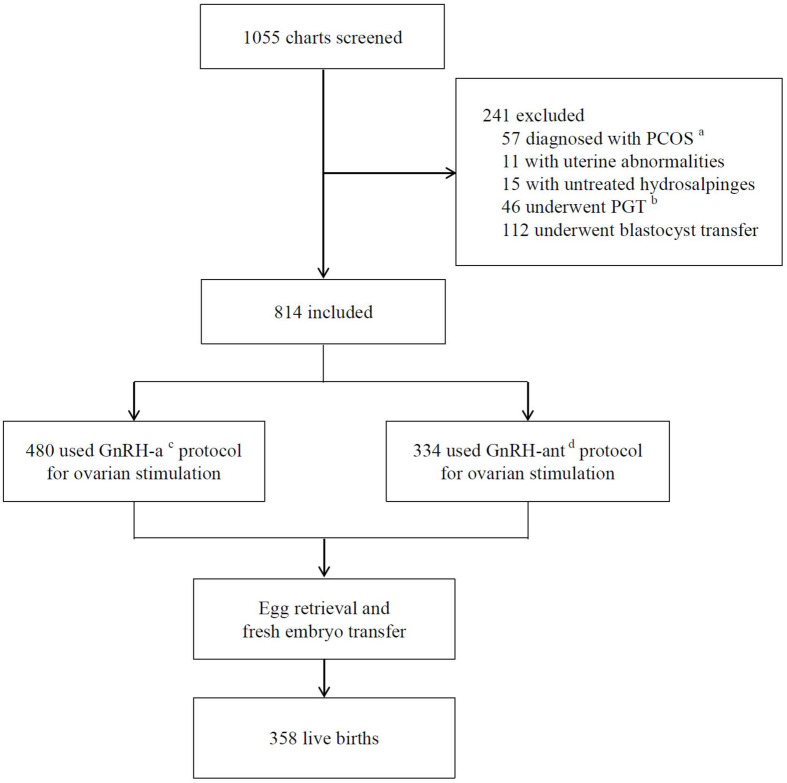
Flow chart of study design. ^a^PCOS-polycystic ovary syndrome. ^b^PGT-preimplantation genetic testing. ^c^GnRH-a-GnRH-agonist long protocol. ^d^GnRH-ant-GnRH antagonist protocol.

A total of 1,055 charts were reviewed for screening, of which 814 patients were included for analysis (Figure 1). The mean age of patients was 28.0 ± 3.2 years with a mean BMI 21.6 ± 2.8 kg/m^2^ and a basal serum FSH 6.7 ± 1.4 IU/L (Table 1). The most common indications for IVF were tubal factor and male factor. 480 (59.0%) patients used the long GnRH-a protocol for ovarian stimulation, while 334 (41.0%) used the GnRH-ant protocol. The mean dose of total Gn was 1,534.1 ± 413.5 IU, and mean number of oocytes retrieved was 10.9 ± 4.1. With an average of 1.9 ± 0.2 cleavage embryos transferred in fresh cycles, the CPR was 51.4%, and 358 (44.0%) patients achieved a live birth.

**TABLE 1:** Baseline characteristics, cycle stimulation characteristics, and pregnancy outcomes of the study population.

	Study population
No. of patients	814
Age (year)	28.0 ± 3.2
Duration of infertility (year)	3.2 ± 2.1
BMI (kg/m^2^)	21.6 ± 2.8
Primary infertility	614 (75.4)
Basal FSH (IU/L)	6.7 ± 1.4
Basal LH (IU/L)	5.1 ± 2.5
Basal Estradiol (pg/ml)	43.2 ± 27.8
Indication for IVF	
Tubal factor only	346 (42.5)
Ovulation dysfunction only	14 (1.7)
Endometriosis only	7 (0.9)
Male factor only	323 (39.7)
Concomitant factors	21 (2.6)
Unexplained	103 (12.7)
Stimulation protocol	
GnRH-a^a^	480 (59.0)
GnRH-ant^b^	334 (41.0)
Total gonadotropin (IU)	1,534.1 ± 413.5
Days of stimulation	9.6 ± 1.4
Estradiol on day of hCG trigger (pg/ml)	2,859.7 ± 1,360.3
Progesterone on day of hCG trigger (ng/ml)	0.9 ± 0.5
Endometrial thickness on day of hCG trigger (mm)	9.8 ± 1.6
ICSI for insemination	236 (29.0)
Number of oocytes retrieved	10.9 ± 4.1
Number of 2PN	7.1 ± 3.3
Number of embryos transferred in fresh cycle	1.9 ± 0.2
Number of embryos cryopreserved	2.7 ± 2.4
Fresh clinical pregnancy rate	418 (51.4)
Fresh early pregnancy loss rate	33 (7.9)
Fresh live birth rate	358 (44.0)
Twin live birth rate	120 (33.5)

**^*a*^GnRH-a-GnRH-agonist long protocol.^*b*^GnRH-ant-GnRH antagonist protocol. Data are shown as mean and standard deviation (M ± SD) or number (percentage).**

Results of multivariate logistic regression analysis demonstrated that using a GnRH-ant protocol was significantly associated with lowered LBR (OR 0.70, 95% CI 0.52–0.93), while endometrial thickness on day of hCG trigger was associated with increased LBR (OR 1.16, 95% CI 1.06–1.27) (Table 2). The AUC for the regression model was 0.59 (*p* < 0.001).

**TABLE 2:** Predictive factors for live birth rate in fresh cycles after OC pretreatment.

	*p*-value	O R(95% CI)
Age (year)	0.16	
Duration of infertility (year)	0.13	
BMI (kg/m^2^)	0.77	
Primary infertility	0.95	
Basal FSH (IU/L)	0.76	
Basal LH (IU/L)	0.32	
Basal Estradiol (pg/ml)	0.66	
Indication for IVF	0.48	
Tubal factor only		1
Ovulation dysfunction only	0.91	
Endometriosis only	0.62	
Male factor only	0.14	
Concomitant factors	0.35	
Unexplained	0.91	
Stimulation protocol	0.013	
GnRH-a^a^		1
GnRH-ant^b^		0.70 (0.52–0.93)
Total gonadotropin (IU)	0.17	
Days of stimulation	0.18	
Estradiol on day of hCG trigger (pg/ml)	0.62	
Progesterone on day of hCG trigger (ng/ml)	0.77	
Endometrial thickness on day of hCG trigger (mm)	0.001	1.16 (1.06–1.27)
ICSI for insemination	0.81	
Number of oocytes retrieved	0.71	
Number of 2PN	0.19	
Number of embryos transferred in fresh cycle	0.23	

**^*a*^GnRH-a-GnRH-agonist long protocol. ^*b*^GnRH-ant-GnRH antagonist protocol. Logistic regression analysis was conducted and data are shown as odds ratio (OR) and 95% confidence interval (CI).**

Based on these findings, we compared patients using GnRH-a and GnRH-ant protocols (Table 3). Patients’ age, duration of infertility, BMI, primary/secondary infertility, basal FSH, basal LH and indication for IVF were all comparable, while basal estradiol was slightly higher in the GnRH-a group. With respect to cycle stimulation characteristics, total Gn dose utilized was significantly higher (1,580.4 ± 408.7 vs. 1,467.7 ± 412.0, *p* < 0.001) and days of stimulation were significantly longer (9.8 ± 1.3 vs. 9.4 ± 1.5, *p* < 0.001) in those using GnRH-a. Comparatively, peak estradiol on the day of hCG trigger (3,097.2 ± 1,400.4 vs. 2,518.4 ± 1,224.1, *p* < 0.001) and endometrial thickness on day of hCG trigger was significantly greater (10.1 ± 1.6 vs. 9.4 ± 1.4, *p* < 0.001) in the GnRH-a group.

**TABLE 3:** Baseline characteristics, cycle stimulation characteristics, and pregnancy outcomes in GnRH-a and GnRH-ant protocols.

	GnRH-a^a^	GnRH-ant^b^	*p*-value
No. of patients	480	334	
Age (year)	27.9 ± 3.1	28.1 ± 3.3	0.39
Duration of infertility (year)	3.2 ± 2.2	3.1 ± 2.0	0.77
BMI (kg/m^2^)	21.6 ± 2.7	21.7 ± 3.1	0.97
Primary infertility	368 (76.7)	246 (73.7)	0.33
Basal FSH (IU/L)	6.7 ± 1.4	6.6 ± 1.4	0.34
Basal LH (IU/L)	5.1 ± 2.5	5.2 ± 2.5	0.43
Basal estradiol (pg/ml)	45.0 ± 28.2	40.7 ± 27.0	0.03
Indication for IVF			0.38
Tubal factor only	205 (42.7)	141 (42.2)	
Ovulation dysfunction only	10 (2.1)	4 (1.2)	
Endometriosis only	6 (1.3)	1 (0.3)	
Male factor only	186 (38.8)	137 (41.0)	
Concomitant factors	15 (3.1)	6 (1.8)	
Unexplained	58 (12.1)	45 (13.5)	
Total gonadotropin (IU)	1,580.4 ± 408.7	1,467.7 ± 412.0	<0.001
Days of stimulation	9.8 ± 1.3	9.4 ± 1.5	<0.001
Estradiol on day of hCG trigger (pg/ml)	3,097.2 ± 1,400.4	2,518.4 ± 1,224.1	<0.001
Progesterone on day of hCG trigger (ng/ml)	0.9 ± 0.6	0.9 ± 0.3	0.20
Endometrial thickness on day of hCG trigger (mm)	10.1 ± 1.6	9.4 ± 1.4	<0.001
ICSI for insemination	143 (29.8)	93 (27.8)	0.55
Number of oocytes retrieved	10.9 ± 4.0	10.9 ± 4.3	0.93
Number of 2PN	7.1 ± 3.3	7.1 ± 3.3	0.93
Number of embryos transferred in fresh cycle	1.9 ± 0.2	1.9 ± 0.3	0.44
Number of embryos cryopreserved	2.7 ± 2.5	2.5 ± 2.4	0.24
Fresh clinical pregnancy rate	273 (56.9)	145 (43.4)	<0.001
Fresh early pregnancy loss rate	24 (8.8)	9 (6.2)	0.35
Fresh live birth rate	233 (48.5)	125 (37.4)	0.002
Twin live birth rate	83 (35.6)	37 (29.6)	0.25

**^*a*^GnRH-a-GnRH-agonist long protocol. ^*b*^GnRH-ant-GnRH antagonist protocol. Data are shown as mean and standard deviation (M ± SD) or number (percentage).**

The number of oocytes retrieved, normal fertilization, and number of embryos transferred were similar between groups; however, the GnRH-ant group had significantly lower CRP (43.4 vs. 56.9%, *p* < 0.001) and LBR following fresh embryo transfer compared to the GnRH-a group (37.4 vs. 48.5%, *p* = 0.002). No differences in EPLR and twin LBR were noted between groups.

**FIGURE 2:**
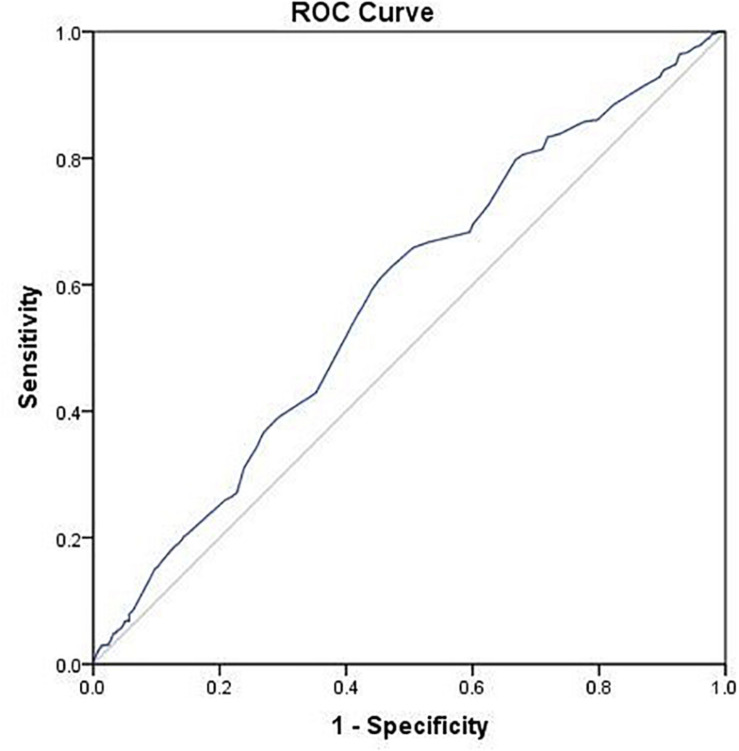
ROC curve of endometrial thickness on the day of hCG trigger for live birth after fresh embryo transfer.

**FIGURE 3:**
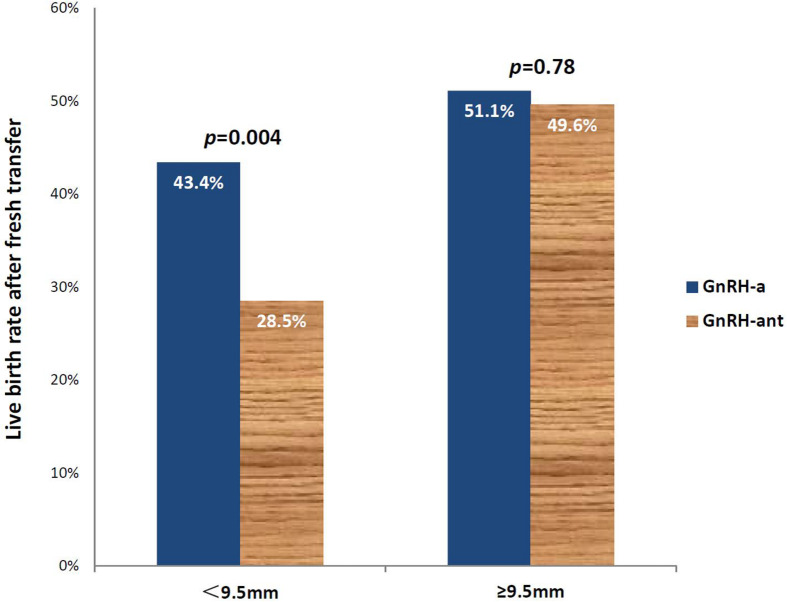
Live birth rates after fresh embryo transfer in those using the GnRH-a^a^ and GnRH-ant^b^ protocols with an endometrial thickness of <9.5 and ≥9.5mm on the day of hCG trigger. ^a^GnRH-a-GnRH-agonist long protocol. ^b^GnRH-ant-GnRH antagonist protocol.

Using ROC analysis, 9.5 mm was identified to be the optimal cut-off point of endometrial thickness on day of hCG trigger with an AUC of 0.58 (*p* < 0.001) (Figure 2 and Table 4). Furthermore, results showed that those with endometrial thickness on the day of hCG trigger <9.5 mm in the GnRH-ant group had a significantly lower CPR (33.7 vs. 56.6%, *p* < 0.001) and LBR (28.5 vs. 43.4%, *p* = 0.004) than the GnRH-a group, while no such differences were noted in those with endometrial thickness on the day of hCG trigger ≥9.5 mm (CPR: 56.7 vs. 57.0%, *p* = 0.96; LBR: 49.6 vs. 51.1%, *p* = 0.78) between the GnRH-ant and GnRH-a group (Figure 3 and Supplementary Table 1).

**TABLE 4:** Predictor of endometrial thickness on day of hCG trigger for live birth after fresh embryo transfer.

Predictor of endometrial thickness	
Area under the receiver operating characteristic curve	0.58
*P*-values	<0.001
Cut-off point (mm)	9.5
Sensitivity	0.63
Specificity	0.53

## Discussion

Our results suggest that in fresh embryo transfer cycles after OC pretreatment, using GnRH-ant protocol for ovarian stimulation was associated with significantly lower LBR (OR 0.70, 95% CI 0.52–0.93), while endometrial thickness on day of hCG trigger was associated with a greater LBR (OR 1.16, 95% CI 1.06–1.27). Using ROC analysis and a cut-off endometrial thickness of 9.5 mm, those <9. 5mm using GnRH-ant resulted in significantly lower LBR than those using a GnRH-a protocol (28.5 vs. 43.4%, *p* = 0.004).

OC are the most popular used pretreatment for IVF cycle scheduling world-wide (Garcia-Velasco and Fatemi, 2015; Pereira et al., 2016; Rodriguez-Purata et al., 2018). It is believed that OC act to suppress endogenous gonadotropin secretion thus preventing spontaneous LH-surge and assist in the synchronization of follicular development when given prior to gonadotropin stimulation, leading to a higher egg yield and increased chances of pregnancy (Kim et al., 2009). However, the results of our study demonstrate lower LBR following fresh embryo transfer in patients using the GnRH-ant protocol for ovarian stimulation, which is consistent with previously published studies (Garcia-Velasco and Fatemi, 2015; Farquhar et al., 2017; Wei et al., 2017; Xu et al., 2019b). Results of a recent meta-analysis of 6 RCTs by Farquhar et al. reported that OC pretreatment was associated with a lower ongoing pregnancy rate/LBR after a fresh embryo transfer (OR 0.74, 95% CI: 0.58–0.95) in GnRH-ant cycles (Farquhar et al., 2017). Secondary analysis of a multicenter RCT including 1,508 women with PCOS using the GnRH-ant protocol also showed that OC pretreatment was significantly associated with lower rate of live birth (OR 0.61, 95% CI: 0.46–0.80). Yet, the impact of OC pretreatment on LBR in fresh GnRH-a cycles is debatable. A retrospective cohort study by Xu et al. (2019b) in 1,025 women using a long GnRH-a protocol found that patients in the OC group resulted in lower LBR than those not (50.5 vs. 59.4%, *p* = 0.45). In contrast, results of a retrospective study by Qin et al. which included 2,052 patients showed that OC had no impact on LBR in the long GnRH-a protocol (59.8 vs. 59.1%, *p* > 0.05) (Xu et al., 2019a).

Endometrial thickness has always appeared to be a predictive factor in successful IVF cycles (Yuan et al., 2016). Results of our study also show that endometrial thickness on day of hCG trigger was positively associated with LBR after fresh transfer (OR 1.16, 95% CI 1.06–1.27). Moreover, patients in the GnRH-ant group appeared to have thinner endometrial thickness which led to decreased LBR. The underlying mechanism regarding potentially impaired endometrial receptivity remains unclear. Some have suggested that OC may induce an advanced endometrium maturation, and together with the effects of ovarian stimulation may magnify the asynchrony between the endometrium and embryos during fresh embryo transfers, thereby resulting in impaired fresh pregnancy outcomes (Creus et al., 2003). However, the known crucial genes expressed during the window of implantation in patients with and without OC pretreatment have failed to detect any relevant changes in gene expression between groups. This fails to explain how OC might impairs endometrial receptivity despite the adverse clinical results (Bermejo et al., 2014).

To the best of our knowledge, few studies have focused on predictive factors after the use of OC pretreatment in fresh IVF cycles. Using good prognosis patients in our study, there appears to be a negative impact of OC on LBR that was most likely associated when endometrial thickness on day of hCG was <9.5 mm in those using GnRH-ant protocols. This suggests that a freeze-all strategy could be considered in this group of patients.

A number of limitations should be noted with our study. Firstly, the nature of retrospective design may lead to selection bias. Although AUCs of ROC curve for multivariate logistic regression model and for endometrial thickness on day of hCG trigger are both significantly different (*p* < 0.001), the predictive ability of both on LBR is limited. Secondly, three types of OC with different components were utilized in this study, and the specific type and duration of OC for each patient was not fully recorded. While there is no definitive evidence to support the difference in the efficacy of different OC (Yildiz, 2015; Xu et al., 2019b), it is difficult to exclude whether different types of OC as well as the duration of use have varied effects on IVF outcomes. Additionally, although result of subgroup analysis showed significant differences in LBR between GnRH-a and GnRH-ant groups with endometrial thickness on the day of hCG < 9.5 mm, the sample size makes conclusions limited. Consequently, well-designed prospective controlled trials are necessary to confirm our results, and generalizability of our results should be considered with caution.

## Conclusion

In conclusion, our results demonstrate that LBR following fresh embryo transfer was significantly associated with the use of GnRH-ant protocol and the endometrial thickness on day of hCG trigger after OC pretreatment, with the use of GnRH-ant being a risk factor, while endometrial thickness is a protective factor. LBR was significantly impacted in OC pre-treated GnRH-ant cycles with an endometrial thickness <9.5 mm on the day of hCG trigger and cryopreservation of all embryos in these cycles should be considered.

## Data Availability Statement

The datasets generated during the current study are available from the corresponding author on reasonable request.

## Ethics Statement

The studies involving human participants were reviewed and approved by the Ethics Committee for Reproductive Medicine of Ren Ji Hospital. The patients/participants provided their written informed consent to participate in this study.

## Author Contributions

YL, YN, YD, XL, SL, and YS performed the material preparation, data collection and analysis. YL, YN, YW, YH, and SL wrote the first draft of the manuscript. All authors contributed to the study conception and design and commented on previous versions of the manuscript, read and approved the final manuscript.

## Conflict of Interest

The authors declare that the research was conducted in the absence of any commercial or financial relationships that could be construed as a potential conflict of interest.

## References

[B1] Bermejo A. Iglesias C. Ruiz-Alonso M. Blesa D. Simón C. Pellicer A. (2014). The impact of using the combined oral contraceptive pill for cycle scheduling on gene expression related to endometrial receptivity. *Hum. Reproduc.* 29 1271–1278. 10.1093/humrep/deu065 24706003

[B2] Bozdag G. Esinler I. Yarali H. (2012). Pretreatment with oral contraceptive pills does not influence the pregnancy rate in the long leuprolide acetate protocol. *Gynecol. Obstet. Invest.* 73 53–57. 10.1159/000329730 22133574

[B3] Creus M. Ordi J. Fábregues F. Casamitjana R. Carmona F. Cardesa A. (2003). The effect of different hormone therapies on integrin expression and pinopode formation in the human endometrium: a controlled study. *Hum. Reproduc.* 18 683–693. 10.1093/humrep/deg177 12660257

[B4] Farquhar C. Rombauts L. Kremer J. A. Lethaby A. Ayeleke R. O. (2017). Oral contraceptive pill, progestogen or oestrogen pretreatment for ovarian stimulation protocols for women undergoing assisted reproductive techniques. *Cochrane Database Syst. Rev.* 5:Cd006109. 10.1002/14651858.CD006109.pub3 28540977 PMC6481489

[B5] Garcia-Velasco J. A. Fatemi H. M. (2015). To pill or not to pill in GnRH antagonist cycles: that is the question! *Reprod. Biomed. Online* 30 39–42. 10.1016/j.rbmo.2014.09.010 25447926

[B6] Golobof A. Kiley J. (2016). The current status of oral contraceptives: progress and recent innovations. *Semin. Reprod. Med.* 34 145–151. 10.1055/s-0036-1572546 26960906

[B7] Keltz M. D. Gera P. S. Skorupski J. Stein D. E. (2007). Comparison of FSH flare with and without pretreatment with oral contraceptive pills in poor responders undergoing in vitro fertilization. *Fertil. Steril.* 88 350–353. 10.1016/j.fertnstert.2006.11.123 17693328

[B8] Kim C. H. Jeon G. H. Cheon Y. P. Jeon I. Kim S. H. Chae H. D. (2009). Comparison of GnRH antagonist protocol with or without oral contraceptive pill pretreatment and GnRH agonist low-dose long protocol in low responders undergoing IVF/intracytoplasmic sperm injection. *Fertil. Steril.* 92 1758–1760. 10.1016/j.fertnstert.2009.05.013 19523618

[B9] Lu W. H. Gu Y. Q. (2010). Insights into semen analysis: a Chinese perspective on the fifth edition of the WHO laboratory manual for the examination and processing of human semen. *Asian J. Androl.* 12 605–606. 10.1038/aja.2010.36 20543855 PMC3739370

[B10] Lu Y. Wang Y. Zhang T. Wang G. He Y. Lindheim S. R. (2020). Effect of pretreatment oral contraceptives on fresh and cumulative live birth in vitro fertilization outcomes in ovulatory women. *Fertil. Steril.* 114 779–786. 10.1016/j.fertnstert.2020.05.021 32741621

[B11] Matsuura K. Hayashi N. Takiue C. Hirata R. Habara T. Naruse K. (2010). Blastocyst quality scoring based on morphologic grading correlates with cell number. *Fertil. Steril.* 94 1135–1137. 10.1016/j.fertnstert.2009.11.003 20079898

[B12] Montoya-Botero P. Martinez F. Rodríguez-Purata J. Rodríguez I. Coroleu B. Polyzos N. P. (2020). The effect of type of oral contraceptive pill and duration of use on fresh and cumulative live birth rates in IVF/ICSI cycles. *Hum. Reprod.* 35 826–836. 10.1093/humrep/dez299 32163564

[B13] Ozgur K. Berkkanoglu M. Bulut H. Humaidan P. Coetzee K. (2016). Agonist depot versus OCP programming of frozen embryo transfer: a retrospective analysis of freeze-all cycles. *J. Assist. Reprod. Genet.* 33 207–214. 10.1007/s10815-015-0639-3 26701802 PMC4759006

[B14] Pereira N. Petrini A. C. Zhou Z. N. Lekovich J. P. Kligman I. Rosenwaks Z. (2016). Pretreatment of normal responders in fresh in vitro fertilization cycles: a comparison of transdermal estradiol and oral contraceptive pills. *Clin. Exp. Reprod. Med.* 43 228–232. 10.5653/cerm.2016.43.4.228 28090462 PMC5234290

[B15] Rodriguez-Purata J. Devesa M. Parriego M. Pardos C. Rodriguez I. Polyzos N. P. (2018). Linking back-to-back stimulation cycles with oral contraceptives or progestins in women undergoing embryo accumulation for preimplantation genetic testing, a retrospective study. *Gynecol. Endocrinol.* 34 955–960. 10.1080/09513590.2018.1473363 29768947

[B16] Rotterdam Eshre/Asrm-Sponsored Pcos Consensus Workshop Group. (2004). Revised 2003 consensus on diagnostic criteria and long-term health risks related to polycystic ovary syndrome. *Fertil. Steril.* 81 19–25. 10.1016/j.fertnstert.2003.10.004 14711538

[B17] Wei D. Shi Y. Li J. Wang Z. Zhang L. Sun Y. (2017). Effect of pretreatment with oral contraceptives and progestins on IVF outcomes in women with polycystic ovary syndrome. *Hum. Reprod.* 32 354–361. 10.1093/humrep/dew325 27999118 PMC5870982

[B18] Xu L. Ding L. Jiang J. Liu P. Wei D. Qin Y. (2019a). Effects of oral contraceptive pretreatment on IVF outcomes in women following a GnRH agonist protocol. *Reprod. Biomed. Online* 39 924–930. 10.1016/j.rbmo.2019.08.002 31680062

[B19] Xu Z. Meng L. Pan C. Chen X. Huang X. Yang H. (2019b). Does oral contraceptives pretreatment affect the pregnancy outcome in polycystic ovary syndrome women undergoing ART with GnRH agonist protocol? *Gynecol. Endocrinol.* 35 124–127. 10.1080/09513590.2018.1500535 30303700

[B20] Yildiz B. O. (2015). Approach to the patient: contraception in women with polycystic ovary syndrome. *J. Clin. Endocrinol. Metab.* 100 794–802. 10.1210/jc.2014-3196 25701301

[B21] Yuan X. Saravelos S. H. Wang Q. Xu Y. Li T. C. Zhou C. (2016). Endometrial thickness as a predictor of pregnancy outcomes in 10787 fresh IVF-ICSI cycles. *Reprod. Biomed. Online* 33 197–205. 10.1016/j.rbmo.2016.05.002 27238372

