# Supplementary material for: Optimal Candidates to Do Fresh Embryo Transfer in Those Using Oral Contraceptive Pretreatment in IVF Cycles
Source: Front Physiol. 2021 Mar 11;12:576917. doi: 10.3389/fphys.2021.576917 (PMC7991902; doi:10.3389/fphys.2021.576917)
Supplement: Supplementary file 1 [file Table_1.DOCX]

**Supplemental table 1**. Pregnancy outcomes after fresh embryo transfer in those using the GnRH-a and GnRH-ant protocols with an endometrial thickness <9.5mm and ≥9.5mm on day of hCG trigger.

|  | **<9.5mm** | | **≥9.5mm** | | **p-value** |
| --- | --- | --- | --- | --- | --- |
|  | **GnRH-a^1^** | **GnRH-ant^2^** | **GnRH-a^1^** | **GnRH-ant^2^** |  |
| No. of patients | 159 | 193 | 321 | 141 |  |
| Age (year) | 28.0±3.1 | 28.2±3.3 | 28.0±3.1 | 28.0±3.4 | 0.80 |
| Fresh clinical pregnancy rate | 90 (56.6) | 65 (33.7) ^a, b^ | 183 (57.0) | 80 (56.7) | <0.001 |
| Fresh early pregnancy loss rate | 13 (14.4) | 4 (6.2) | 11 (6.0) | 5 (6.3) | 0.08 |
| Fresh live birth rate | 69 (43.4) | 55 (28.5) ^a, b^ | 164 (51.1) | 70 (49.6) | <0.001 |
| Twin live birth rate | 22 (31.9) | 15 (27.3) | 61 (35.5) | 22 (31.4) | 0.54 |

^1^ GnRH-a-GnRH-agonist long protocol. ^2^ GnRH-ant-GnRH antagonist protocol. Data are shown as mean and standard deviation (M±SD) or number (percentage)

^a^ *p*<0.05 in GnRH-a vs. GnRH-ant when <9.5mm; ^b^ *p*<0.05 when <9.5 vs. ≥9.5mm in GnRH-ant
